# Comparative evaluation of different medication safety measures for the emergency department: physicians’ usage and acceptance of training, poster, checklist and computerized decision support

**DOI:** 10.1186/1472-6947-13-79

**Published:** 2013-07-29

**Authors:** Brita Sedlmayr, Andrius Patapovas, Melanie Kirchner, Anja Sonst, Fabian Müller, Barbara Pfistermeister, Bettina Plank-Kiegele, Renate Vogler, Manfred Criegee-Rieck, Hans-Ulrich Prokosch, Harald Dormann, Renke Maas, Thomas Bürkle

**Affiliations:** 1 Chair of Medical Informatics, University Erlangen-Nuremberg, Krankenhausstraße 12, 91054, Erlangen, Germany; 2 Klinikum Fuerth, Fuerth, Germany; 3 Institute of Experimental and Clinical Pharmacology, University Erlangen-Nuremberg, Nuremberg, Germany

**Keywords:** Evaluation, Medication safety, Emergency department, Clinical decision support systems, Technology acceptance, TAM2, Patient safety

## Abstract

**Background:**

Although usage and acceptance are important factors for a successful implementation of clinical decision support systems for medication, most studies only concentrate on their design and outcome. Our objective was to comparatively investigate a set of traditional medication safety measures such as medication safety training for physicians, paper-based posters and checklists concerning potential medication problems versus the additional benefit of a computer-assisted medication check. We concentrated on usage, acceptance and suitability of such interventions in a busy emergency department (ED) of a 749 bed acute tertiary care hospital.

**Methods:**

A retrospective, qualitative evaluation study was conducted using a field observation and a questionnaire-based survey. Six physicians were observed while treating 20 patient cases; the questionnaire, based on the Technology Acceptance Model 2 (TAM2), has been answered by nine ED physicians.

**Results:**

During field observations, we did not observe direct use of any of the implemented interventions for medication safety (paper-based and electronic). Questionnaire results indicated that the electronic medication safety check was the most frequently used intervention, followed by checklist and posters. However, despite their positive attitude, physicians most often stated that they use the interventions in only up to ten percent for subjectively “critical” orders. Main reasons behind the low usage were deficits in ease-of-use and fit to the workflow. The intention to use the interventions was rather high after overcoming these barriers.

**Conclusions:**

Methodologically, the study contributes to Technology Acceptance Model (TAM) research in an ED setting and confirms TAM2 as a helpful diagnostic tool in identifying barriers for a successful implementation of medication safety interventions. In our case, identified barriers explaining the low utilization of the implemented medication safety interventions - despite their positive reception - include deficits in accessibility, briefing for the physicians about the interventions, ease-of-use and compatibility to the working environment.

## Background

Medication errors and adverse drug reactions (ADRs) are one of the most frequent causes of death in many countries [1-3]. In Germany, between 17.000 and 58.000 deaths per year may be caused by medication errors [4,5]. Up to 50 percent of these cases are judged to be preventable. Prevention strategies include medication safety guidelines, improved training and awareness of staff as well as computerized clinical decision support systems (CDSS). Such strategies promise to reduce medication errors and adverse drug events [6-9], but without guaranteeing success. Improved pharmacotherapy implies that clinicians apply those tools and utilities on a regular basis. Several studies demonstrated that computerized CDSS were not successful due to limited or no system use [10-14]. The launch of such systems requires an adequate awareness and the determination to alter existing medication procedures, thus making user acceptance a key factor for successful implementation [15]. Several methods and models have been published to examine factors which influence the acceptance of innovations. Examples include the Theory of Reasoned Action (TRA) [16,17], the Theory of Planned Behavior (TPB) [18], the Task-Technology Fit Model [19,20], the Diffusion of Innovation (DOI) Theory [21] or the Technology Acceptance Model (TAM) [22] with its extensions TAM2 [23], TAM3 [24] and the Unified Theory of Acceptance and Use of Technology (UTAUT) [25]. In the clinical context TAM (respectively its extensions) is used increasingly [26,27]. While TAM postulates that perceived usefulness and perceived ease-of-use are the key factors in the adoption, its further development TAM2 posits subjective norm, image, job relevance, output quality and result demonstrability as additional antecedents to the perceived usefulness variable. TAM3 advanced TAM2 with six additional antecedents for the perceived ease-of-use variable, which are computer self-efficacy, perceptions of external control, computer anxiety, computer playfulness, perceived enjoyment and objective usability. The UTAUT has integrated different technology acceptance models including TAM and contains performance expectancy (equivalent to perceived usefulness in TAM), effort expectancy (equivalent to perceived ease-of-use in TAM), social influence and facilitating conditions as core predictors.

In a research project supported by the German Federal Ministry of Health, drug associated risk situations are analyzed among patients visiting the emergency department (ED) of a tertiary care acute hospital in Germany [28,29]. Interventions included different paper- and computer-based medication safety measures, which were implemented in the ED. The primary aim of this research was:

1. to examine whether and to what extend the different interventions were used

2. to assess the acceptance and suitability of the different medication safety interventions for the ED based on the acceptance model TAM2 and

3. to detect reasons for adoption or rejection of interventions and to derive recommendations for enhancing acceptance and use of interventions.

## Methods

### Study design and setting

A retrospective, qualitative study was conducted following the introduction of a series of interventions to improve medication safety. In a first step a field observation was carried out. In a second step a questionnaire, based on the TAM2 framework [23], was deployed to assess use and acceptance of the different medication safety interventions.

The study was conducted at the ED of Klinikum Fuerth, Germany, a tertiary care hospital with 749 beds and an annual ED census of 45.000 patients. During the time of the interventions the ED team comprised one consultant, three senior physicians, two specialists in internal medicine and six junior doctors. In 2011 a set of paper-based and computer-based interventions have been implemented consecutively in the ED to improve medication safety within a grant project from the “German Coalition for Patient Safety” (BMG grant II A 5–2509 ATS 003) [30].

The ethics board of the Friedrich-Alexander-University of Erlangen-Nuremberg, Germany reviewed and approved the study protocol. The Fuerth data safety commissioner reviewed the technical infrastructure, data handling and pseudonymization procedures. The Fuerth staff council approved the participation of the employees.

### The interventions to improve medication safety

All medication safety interventions were implemented previous to this study. Paper-based interventions included repeated training, posters concerning typical medication safety problems and a pocket checklist listing critical drugs and symptoms. Electronic interventions resulted in a CDSS application [31] integrated with the hospital’s electronic health record (EHR) system [32].

#### Repeated training

Along with physicians’ Monday rounds, additional regular medication safety training sessions were implemented to increase physicians’ awareness of medication hazards such as critical drug-drug interactions (DDIs), medication errors (MEs) and recent safety warnings of the drug agency and regulatory authorities. Physicians practiced the basic strategies for the recognition and prevention of adverse drug events and were trained to use available public domain drug information systems in the Internet. Furthermore periodical quality circles with case review were carried out.

#### Paper posters and pocket checklists

To support physicians, information posters and checklists were developed comprising lists of potentially inappropriate medications in order to quickly identify patients of high risk. Lists for posters and checklists were conceptually similar and included critical drugs, typical drug associated symptoms, potential alternative drugs and supportive procedures. The posters were displayed prominently in the ED main room. The checklists were intended for physicians’ white coat pocket and were distributed to all physicians working in the ED.

#### Computerized decision support

Initially neither computerized drug order entry (cPOE) nor electronic CDSS were established in the ED. Prior to this evaluation study, in 2011, the commercial EHR system of the hospital [32] was modified and an electronic ED case sheet was implemented to enable cPOE. In addition, a commercially available CDSS for medication safety checks [31] with an embedded drug information system (DIS) [33] was integrated into the EHR system. As a first measure, an electronic infobutton was implemented to show brief information regarding critical contraindications and DDIs similar to the paper-based checklist. Additional electronic measures included one checkbox for each drug to prompt the physician if the drug had a valid indication based on the patient status, one checkbox for the patient case sheet itself to prompt the physician if an adverse drug event (ADE) was likely and finally a “MediCheck” button to send patient data to the commercial CDSS and DIS for a comprehensive workup [34]. The new electronic case sheet with these medication safety functions was made available at all five work stations in ED (three stationary PCs located in the resuscitation room and two computers on wheels). The use of electronic documentation was not mandatory; the previously existing paper-based case sheet could be used alternatively.

### The field observation

Targeted participants of the observation were physicians working permanently or temporarily in shifts in the ED. The sample size was determined by the selected observation dates. Six observations took place in the main resuscitation room in April and May 2012 (three in the morning, three in the afternoon), over a period of five weeks. Each visit lasted approximately two hours, physicians were observed by a trained observer.

Observational results were recorded in semi-structured pseudonymized notes, containing the job status of the observed physician, the type of case sheet used (paper or electronic form), the number of (critical) drugs entered in the electronic case sheet and which measures (poster, checklist and/or digital case sheet) were applied. For the electronic interventions the type of computer-assisted check (infobutton, checkbox “indication for drug known”, checkbox “adverse drug event”, “MediCheck”-button) was recorded. In addition, environmental specifics, e.g. system failures, lack of accessibility or work interruptions were documented. System logs were used to cross-check the observation results and to generate usage statistics for the observation days. During analysis, the data has been completely anonymized. Observation results were summarized in tabular format using a thematic coding frame and the results have been analyzed descriptively.

### The TAM2 based questionnaire

Following the field observation a paper-based questionnaire was distributed to the 12 physicians belonging to the permanent ED staff in May 2012 during a regular physician meeting and recollected at the end of the meeting. Participation was voluntarily and anonymity was guaranteed. The questionnaire instrument had three partitions:

● Part A examined the use of the different medication safety interventions and included open ended questions to determine reasons for restricted use.

● Part B was based on the TAM2 research model and comprised questions on physicians’ acceptance as well as the rated suitability of the measures used.

● Part C concerned basic demographic data such as job status, working experience and computer skills.

The complete questionnaire is available as Additional file 1. The questionnaires were analyzed descriptively for frequency, means, and standard deviations. For all statistic evaluations cases with missing values were listwise deleted. Exact Wilcoxon signed-rank test was used to compare acceptance data between paper-based and electronic-based measures. Pearson correlation coefficients were determined for relations between acceptance variables. In order to account for small sample sizes Monte Carlo approximations with 10.000 samples were applied for significance testing. Friedman test was applied for testing differences between measures’ suitability rankings. All statistics were performed at a 5 percent significance level. All calculations were conducted via SPSS 20.0.

The TAM2 research model applied in part B has been used previously in the medical field (e.g. [35-38]). TAM2 can be used to determine reasons why a new technology is adopted or declined. In our case, TAM2 was applied as well for paper-based interventions and for computerized interventions. According to TAM2, user acceptance - measured by the intention to use a technology - is depending on perceived ease-of-use and perceived usefulness. “Perceived ease-of-use” (PEOU) refers to the degree a person believes that using the technology requires effort. “Perceived usefulness” (PU) is defined as the degree a person beliefs the technology enhances his/her job performance. Then, there are three cognitive factors (job relevance, output quality, result demonstrability) and three social factors (subjective norm, image, voluntariness) which influence usage intention (ITU) indirectly. Among those, “Job relevance” (JR) is defined as individual’s perception of the degree to which technology is relevant for his/her job. “Output quality” (OQ) is defined as individual’s perception of how well a technology performs tasks necessary in his/her job. “Result demonstrability” (RD) describes tangibility of results using the technology. “Subjective norm” (SN) is the degree to which an individual perceives that important others believe he/she should (not) use the technology. “Image” (IM) is defined as the degree to which one perceives the use of the technology as means to enhance the own social status. “Voluntariness” (VO) describes if the decision to adopt the technology is self-determined. In addition, the relationship between “perceived ease-of-use” (PEOU) and “usage intention” (ITU) is moderated by the factor “experience” (EXP), whereas TAM2 postulates that with increasing experience the effect of PEOU and ITU will decrease.

**Figure 1:**
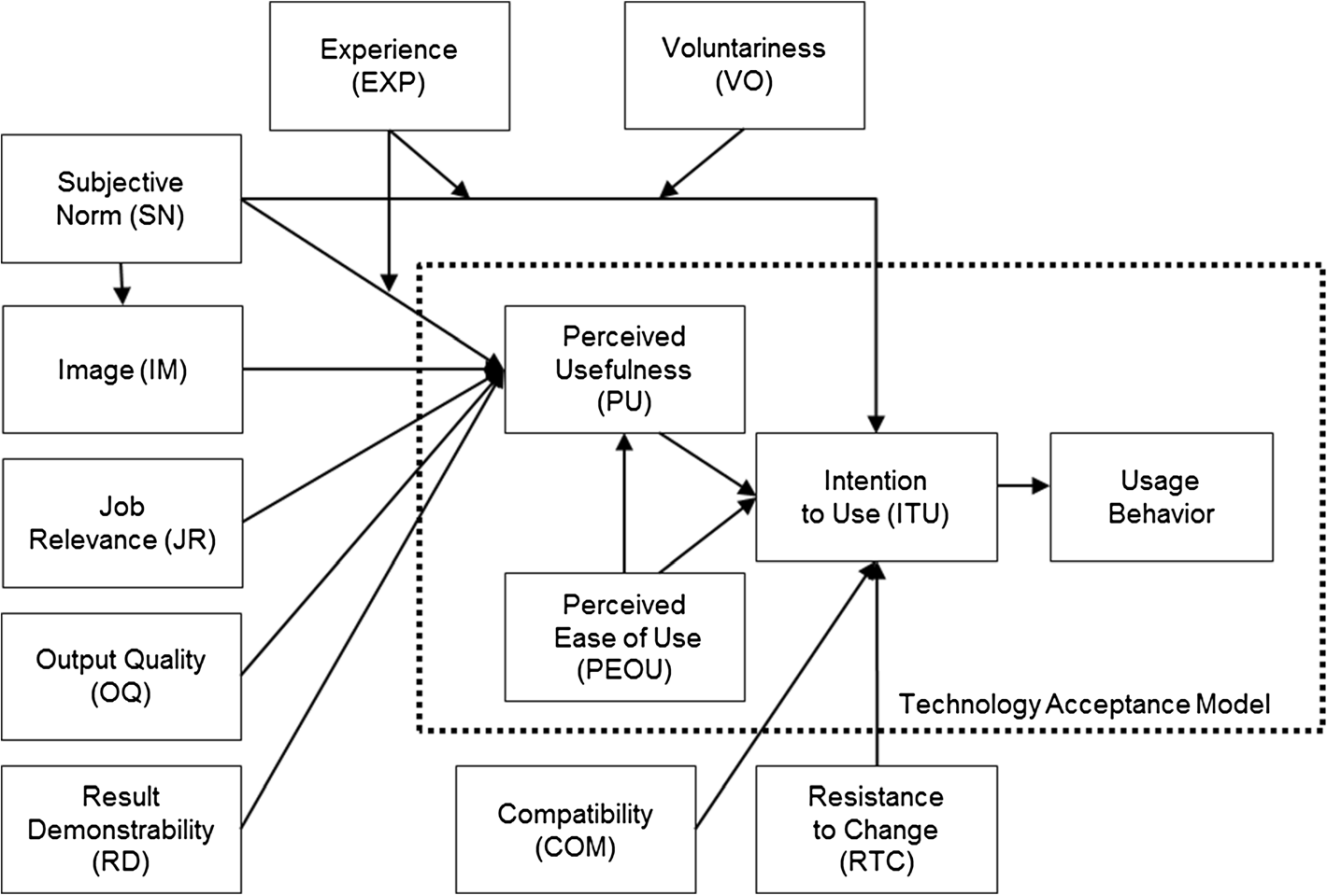
Adapted TAM2 model.

Although TAM2 is an established model, it needed extension with further constructs to capture the unique contextual features and to enhance its explanation capabilities for the ED medication safety field. Based on the previous field observation and suggestions from literature [39,40] TAM2 was extended with the variables “compatibility” (to workflow) defined as “the degree to which an innovation is perceived being consistent with the existing values, needs, and past experiences” [40] and “resistance to change”, described as “any conduct that serves to maintain the status quo in the face of pressure to alter the status quo” [39]. The extended TAM2 used here to explain the adoption or rejection of implemented medication safety interventions is presented in Figure 1.

The TAM2 scales were operationalized using validated items from previous research. Items for “perceived usefulness” (PU), “perceived ease-of-use” (PEOU), “subjective norm” (SN), “image” (IM), “job relevance” (JR), “output quality” (OQ), “result demonstrability” (RD) and “intention to use” (ITU) were taken from the validated questionnaire of Chismar and Wiley-Patton [35]. This questionnaire was developed for future internet-based health care applications in a pediatric community and needed adaptations such as translation into German language, changes in wording (“Internet-based applications” was replaced with “medication safety measures”; “pediatric care” was replaced with “patient care”) and changes of future tense to present tense in the items. The additional items to examine physicians’ “resistance to change” (RTC), “compatibility” (COM) to work practice and “voluntariness of use” (VO) have been added from Bhattacherjee and Hikmet [39], Moore and Benbasat [40] and Venkatesh and Davis [23]. Response to all TAM2 items was measured on a five-point Likert scale ranging from 1 = strongly disagree to 5 = strongly agree. To avoid enforced decisions and distortion of the results the option “not applicable” was available.

The modified questionnaire was reviewed by two experts in the field of health IT evaluation and one medical expert in the field of acute care. In addition, the second part of the questionnaire was piloted with nine employees of the University Medical Centre Erlangen-Nuremberg. Based on participants’ feedback, wording was slightly modified and four items were removed. The resulting Cronbach’s Alpha values (ranging from 0.86 to 1.00) indicated a satisfactory reliability level. The final TAM2 part included 26 items (see Table 1).

**Table 1:** Summary of the measurement items

**Items**	**Measurement**	**Cronbach’s α after item deleted**	**Source**
U1	If you have questions on interactions and contraindications of drugs: How do you inform yourself at first? (look at the poster/look at the pocket- checklist/use the electronic medication check/ask colleagues/look at the “red list” (print version)/others -please specify)	--	self- development
U2	Have you gotten a briefing regarding the usage of the measures? (yes/no)		
U3	How often do you use the measures in your daily routine? (not at all/monthly/weekly/once a day/several times a day)		
U4	In case of a critical drug order: To what percentage do you use the measures? (not at all/up to 10%/up to 25%/up to 50%/up to 100%)		
U5	When have you used the measure for the last time? (never use/today/…days before)		
U6	Which information sources do you usually use when checking medication electronically? (infobutton/checkbox “indication for drug known”/OntoDrug/PharmIndex/checkbox “ADE”/use no source)		
U7	In which situations do you use the computer-assisted medication check? (open- ended)		
U8	Previous field observations indicated that measures have not been used often. What can be the reasons for low usage in your opinion? (open-ended)		
U9	What should be improved so that measures are used more frequently? (open-ended)		
**PU**		**0**.**882**	PU1- self- development; [35]
PU1	Medication safety measures basically make sense.		
PU2	The measures could increase my productivity.		
PU3	The measures could improve the quality of care.		
PU4	The measures could enhance my effectiveness.		
PU5	The measures could be useful in my job.		
**PEOU**		**0**.**949**	[35]
PEOU1	My interaction with measures is clear and understandable.		
PEOU2	The measures are easy to use.		
PEOU3	Interacting with measures will not require a lot of mental effort.		
PEOU4	It will be easy to get measures to do what I want them to do.		
**SN**		**--**	[35]
SN1	Physicians who influence my behavior think I should use the measures.		
**IM**		**0.881**	[35]
IM1	Having the measures is a status symbol.		
IM2	Physicians who use the measures have more prestige than those who do not.		
IM3	Physicians who use the measures have a high profile.		
**JR**		**0**.**869**	[35]
JR1	Usage of the measures is relevant to the delivery of patient care.		
JR2	Usage of the measures is important to the delivery of patient care.		
**OQ**		**--**	[35]
OQ1	The quality of drug information is high.		
**RD**		**0**.**860**	[35]
RD1	The measures could reduce the costs of my care delivery.		
RD2	I believe I can communicate to others the consequences of using the measures.		
RD3	The results of using the measures are apparent to me.		
RD4	I have difficulty explaining why using the measures may or may not beneficial.		
**COM**		--	[40]
COM1	Using the measures fits well with the way I work.		
**RTC**		**0**.**860**	[39]
RTC1	I don’t want the measures to change the way I order patient medication.		
RTC2	Overall, I don’t want the measures to change the way I currently work.		
**VO**		--	[23]
VO	Use of the measures is voluntary.		
**ITU**		**1**.**000**	[35]
ITU1	Assuming that significant barriers to the use are overcome, I intend to use the measures.		
ITU2	If significant barriers did not exist, I predict I would use the measures.		
**General Suitability**			
GLO1	How suitable do you judge the measures (training/poster/checklist/infobutton/checkbox “indication for drug known”/OntoDrug/PharmIndex/checkbox “ADE”) for emergency department? (5-point rating scale ranging from 1 = least suitable to 5 = above all suitable)	--	self- development
DG1	What is your professional status? (senior physician/specialist in internal medicine/junior doctor)	--	self- development
EXP1	How long have you been working as a physician? (for…years)		
EXP2	How long have you been working with electronic patient records? (for…years)		
EXP3	How do you rate your computer skills? (low/moderate/high)		

## Results

### Field observation results

From April to May 2012 six observations were conducted and 20 cases involving a total of six physicians were observed. ED physicians of permanent staff were observed in 60% (12/20) of the cases and physicians working temporarily at the ED in 40% (8/20) of the cases.

In 90% (18/20) of the observed cases the electronic case sheet was used for documenting, but only in 22% (4/18) of these cases detailed drug information for each drug was entered into the electronic case sheet. In none of the cases utilization of the paper-based interventions (pocket checklist or poster) could be observed. Whenever drugs were associated with an infobutton indicating that this drug was involved in a drug risk situation, this was ignored in all cases and the button was not invoked. Furthermore, no utilization of the checkbox “indication for drug known” could be observed. In none of the observed cases the physicians selected the comprehensive “MediCheck” CDSS and DIS call (Table 2).

**Table 2:** Results of field observation on the use of the interventions

**Case**	**Status of the observed person**	**Type of case sheet**	**Medication according to…**	**Documented drugs**	**Drugs with infobutton**	**Used interventions**
1	specialist in internal medicine ED	digital	annex	0	.	none
2	specialist in internal medicine ED	digital	annex	0	.	none
3	specialist in internal medicine ED	digital	annex	0	.	none
4	specialist in internal medicine ED	digital	annex	0	.	none
5	specialist in internal medicine ED	digital	annex	0	.	none
6	junior doctor Med1/Med2	digital	.	6	2	none
7	junior doctor Med1/Med2	digital	annex	0	.	none
8	junior doctor Med1/Med2	digital	medication plan of family physician	8	3	none
9	junior doctor Med1/Med2	digital	medication plan of family physician	8	5	none
10	junior doctor Med1/Med2	digital	annex	0	.	none
11	junior doctor Med1/Med2	digital	.	.	.	none
12	specialist in internal medicine ED	digital	.	2	0	none
13	specialist in internal medicine ED	digital	annex	0	.	none
14	specialist in internal medicine ED	digital	annex	0	.	none
15	junior doctor ED	digital	annex	0	.	none
16	senior physician ED	digital	patients statements	0	.	none
17	medical specialist Med1/Med2	paper	.	.	.	none
18	junior doctor ED	digital	patients statements	.	.	none
19	medical specialist Med1/Med2	paper	patients statements	.	.	none
20	junior doctor ED	digital	.	.	.	none

*Notes*: “.” = *unobservable events.*

A specific cross-check of the electronic case sheets for all six observation days identified that within the six times 24 hours 62% of the electronic case sheets (126/204) were without recorded drugs. 17.5% of these cases (22/126) received de facto no drugs. But in 65% (82/126) the checkbox “medication according to annex” (a choice on the digital case sheet) was selected instead of entering the drugs into the system. Thus, in the majority of cases patients’ medication was only recorded in the paper case sheet or the paper medication list obtained from the family physician or nursing home was simply attached to the paper case sheet. In those cases the corresponding drug indications or contraindications could not be checked electronically.

### Questionnaire results

#### Subject characteristics (part C)

Ten responses were obtained from a total of 12 physicians. A first analysis of the responses showed that one participant missed a substantial portion of the questionnaire and therefore was excluded from later analysis. Nine valid questionnaires with an absolute response rate of 75 percent were included. Among them there were two senior physicians, one specialist in internal medicine and six junior physicians. Age and gender have not been asked. About two-thirds (6/9) of the respondents had less than five years medical experience. Half of the participants (5/9) reported “less than 3 years” experience with electronic medical records; the majority (7/9) judged their computer experience “medium”. Seven physicians had used the paper-based interventions in the past, eight physicians had used the computer applications in the past and one physician had no experiences with either intervention.

#### Self-reported use of the interventions (part A)

44.4% (4/9) of the participants preferred the usage of the electronic interventions over paper-based checklist (11.1%, 1/9) and posters (0/9), while looking for information about contraindications and drug-drug interactions (item U1). In addition, two physicians used the “Arzneimittel pocket” (popular German pocket drug reference), one respondent indicated relying on the Internet and one physician mentioned asking colleagues.

We included two items for assessing utilization of medication safety interventions. Table 3 illustrates the results of the first item “frequency of use” (item U3), which was measured on a five-point scale ranging from “don’t use it at all” to “several times a day”. We summarized “once a day” and “several times a day” under the category “daily”. As results show, the electronic medication safety check (8 users) was reportedly the most frequently used intervention compared to the paper-based checklist (6 users) and poster (4 users). More than half of the participants (5/9) reported daily use of the electronic interventions; one third (3/9) indicated weekly use. Narrative comments (item U7) indicated that the check is mostly applied (a) during patient admission, (b) when reading the case sheet, (c) before ordering drugs for checking dosage, (d) before transferring patients to another ward and (e) in all situations of polypharmacy. In detail (item U6), the infobutton was the most widely utilized electronic function (8/8 physicians), followed by the “MediCheck” invoked DIS and CDSS (5/8 physicians each). The checkbox “adverse drug event” was reportedly used by half (4/8) of the physicians, the checkbox “indication for drug known” was used least (2/8 physicians).

**Table 3:** Frequency of use of the interventions in daily routine

	**Response categories**				
**Interventions**	**No usage**	**Monthly**	**Weekly**	**Daily**	**No answer**
**Paper poster**	4	1	1	2	1
**Paper checklist**	2	3	0	3	1
**Digital case sheet**	1	0	3	5	0

Number of physicians who agreed to the specific category.

The second item “In case of a critical drug order: To what percentage do you use the measures?” (item U4) was most frequently answered with “up to 10%” for all types of paper-based and computerized interventions. However, we identified two participants (number 4 and 8), who applied all interventions for “up to 50%” respectively “up to 100%” of the critical orders (Table 4). Almost one quarter of the participants stated that they got no briefing for the use of posters, checklists and electronic medication checks (item U2).

**Table 4:** Frequency of use in situations of subjectively “critical” drug orders

**Physician**	**Paper poster**			**Paper checklist**			**Digital case sheet**		
	**Got briefing**	**Frequency of use**	**Usage in …% of critical orders**	**Got briefing**	**Frequency of use**	**Usage in …% of critical orders**	**Got briefing**	**Frequency of use**	**Usage in …% of critical orders**
1	n.a.	n.a.	n.a.	n.a.	n.a.	n.a.	yes	weekly	up to 10%
2	yes	monthly	up to 10%	yes	monthly	up to 10%	yes	weekly	up to 10%
3	no	never	--	no	never	--	yes	weekly	up to 25%
4	yes	weekly	up to 25%	no	several times a day	up to 50%	no	several times a day	up to 50%
5	yes	once a day	up to 10%	yes	several times a day	up to 25%	yes	several times a day	up to 25%
6	yes	never	--	yes	monthly	never	yes	once a day	up to 10%
7	yes	never	--	yes	monthly	up to 10%	yes	once a day	up to 50%
8	yes	once a day	up to 50%	yes	several times a day	up to 100%	yes	several times a day	up to 100%
9	no	never	--	no	never	--	no	never	--

*Notes*: „*n*.*a*.” = *no answer.*

With item U8 we asked for reasons for the low utilization previously identified in the field observation. There, the participants named (a) time effort, (b) availability, (c) missing briefing for the interventions, (d) (missing) ease-of-use and (e) (insufficient) familiarization as main barriers. One of the main reasons for not using posters was the lack of availability at the point of care as they were not visible from all treatment cubicles. For the checklists physicians reported missing briefing and low awareness as they were better accustomed to the popular “Arzneimittel pocket” drug reference. Also, the checklist was considered too specific by only providing a limited list of critical drugs. The most important reason to omit the electronic medication safety interventions was lack of time to record all patients’ admission drugs manually in the system. Furthermore, physicians indicated some usability problems e.g. to document half tablets in the case sheet. Corresponding to these answers, the participants suggested improved briefing, faster drug documentation and the need for more time to adapt to the interventions in order to improve utilization (item U9).

#### Acceptance of the interventions according to TAM2 (part B)

Data is based on answers of those physicians, who used the medication safety interventions in the past: n = 8 for the computer-based interventions (functionalities of the digital case sheet), n = 7 for the paper-based interventions training, paper-based posters and pocket checklist. Table 5 presents the average ratings of the different interventions for each construct. Although ratings for paper-based interventions were mostly somewhat lower, differences between computer and paper-based interventions were not significant due to the small sample size (exact Wilcoxon signed-rank test, p < 0.05).

**Table 5:** **Mean scores of physicians’ perceptions of the paper**-**based** (“**paper**”) **and computer**-**based** (“**IT**”) **interventions** (**five**-**point**-**scale**: **1** = **strongly disagree**; **5** = **strongly agree**)

	**Physician**									
**Construct**	**1**	**2**	**3**	**4**	**5**	**6**	**7**	**8**	**Mean**	**SD**
PU^paper^	2.67	4.40	--	2.80	4.40	1.60	4.60	4.80	**3**.**61**	1.240
PU^IT^	2.67	5.00	4.20	4.80	5.00	4.40	4.60	4.80	**4**.**43**	0.767
PEOU^paper^	2.00	3.00	--	4.50	3.75	2.00	4.75	3.50	**3**.**36**	1.098
PEOU^IT^	2.00	5.00	4.00	3.00	3.75	4.00	2.50	2.75	**3**.**38**	0.982
SN^Paper^	.	3.00	--	5.00	5.00	.	4.00	1.00	**3**.**60**	1.673
SN^IT^	.	4.00	5.00	5.00	5.00	.	4.00	1.00	**4**.**00**	1.549
IM^paper^	.	2.50	--	3.00	2.50	3.00	3.00	3.00	**2**.**83**	0.258
IM^IT^	.	3.50	3.50	3.00	2.50	3.00	3.00	3.00	**3**.**07**	0.345
JR^paper^	4.50	3.00	--	4.00	4.00	5.00	5.00	5.00	**4**.**36**	0.748
JR^IT^	4.50	5.00	4.00	4.00	4.00	5.00	5.00	5.00	**4**.**56**	0.496
OQ^paper^	.	3.00	--	3.00	.	5.00	3.00	5.00	**3**.**80**	1.095
OQ^IT^	.	5.00	3.00	5.00	.	5.00	3.00	5.00	**4**.**33**	1.033
RD^paper^	3.00	3.25	--	3.00	4.00	3.00	4.00	5.00	**3**.**61**	0.762
RD^IT^	3.00	4.50	3.50	4.25	4.00	5.00	4.00	5.00	**4**.**16**	0.694
COM^paper^	2.00	2.00	--	2.00	4.00	1.00	4.00	3.00	**2**.**57**	1.134
COM^IT^	2.00	5.00	3.00	4.00	4.00	4.00	4.00	2.00	**3**.**50**	1.069
¬RTC^paper^	4.00	4.50	--	.	4.00	5.00	5.00	5.00	**4**.**58**	0.492
¬RTC^IT^	4.00	4.50	3.00	.	4.00	5.00	5.00	5.00	**4**.**36**	0.784
VO^paper^	5.00	5.00	--	5.00	3.00	5.00	5.00	4.00	**4**.**57**	0.787
VO^IT^	5.00	5.00	4.00	5.00	3.00	5.00	5.00	2.00	**4**.**25**	1.165
ITU^paper^	2.50	4.00	--	5.00	.	1.00	5.00	.	**3**.**50**	1.732
ITU^IT^	2.50	5.00	4.00	5.00	.	5.00	5.00	.	**4**.**42**	1.021

All values non-significant in the exact Wilcoxon signed-rank test. Meaning of abbreviations: *PU*, perceived usefulness; *PEOU* perceived ease-of-use; *SN* subjective norm; *IM* image; *JR* job relevance; *OQ* output quality; *RD* result demonstrability; *COM* compatibility; *RTC* resistance to change; *VO* voluntariness; *ITU* intention to use.

*Notes*: “¬RTC” = *values of RTC were reverse coded*; “.” = *could not be calculated because of missing values*; “--“= *interventions were not used.*

Overall, the results showed a moderate acceptance of the various measures. Most of the mean values were around the midpoint of 3 (“partly agree”) or achieved a rating of 4 (“rather agree”) for the paper-based respectively information technology-based (IT) interventions. The lowest rated factors were image (IM, paper: 2.83/IT: 3.07), compatibility to the work flow (COM, paper: 2.57/IT: 3.50) and perceived ease-of-use (PEOU, paper: 3.36/IT: 3.38). The highest rated factors were job relevance (JR, paper: 4.36/IT: 4.56), the voluntariness of use (VO, paper: 4.57/IT: 4.25) and (no) resistance to change (¬RTC, paper: 4.58/IT: 4.36).

Both, paper-based and IT-based interventions were rated as useful (PU, paper: 3.61/IT: 4.43), important to support the work (JR, paper: 4.36/IT: 4.56) and were of good information quality (OQ, paper: 3.80/IT: 4.33). The results of the medication safety measures were “tangible”, observable and communicable (RD, paper: 3.61/IT: 4.16). The influence of colleagues on the usage behavior was judged as moderate (SN, paper: 3.60/IT: 4.00). The application of the measures was assessed as own free will (VO, paper: 4.57/IT: 4.25). The perceived ease-of-use (PEOU, paper: 3.36/IT: 3.38) was ranked as moderate. The respondents did not confirm that the use of such measures will improve one’s status (IM, paper: 2.83/IT: 3.07). Although the application of the interventions did not fit into their current workflow (COM, paper: 2.57/IT: 3.50), physicians were willing to adapt their way of working (¬RTC, paper: 4.58/IT: 4.36). The intention to use the measures was moderate for paper-based interventions (ITU, paper: 3.50) and rather high for IT-based interventions (ITU, IT: 4.42) if barriers would be overcome.

#### Relationship between TAM2 variables

To examine the relationship between variables and to determine whether “perceived usefulness” (PU) and “perceived ease-of-use” (PEOU) are associated with the “intention to use” (ITU) and self-reported usage a correlation analysis was conducted. Due to the small sample size and the use of the more rigid Monte Carlo testing for small samples most of these relationship tests did not deliver statistically significant values. We could however demonstrate a strong positive correlation between PU and ITU for computer-based interventions (r = 0.960, p < 0.05). Thus, the more useful the system was perceived the more likely the participants would use it. The correlation between PEOU and ITU was moderately positive (r = 0.520), but not significant. A strong positive correlation was found between COM and ITU (r = 0.917, p < 0.05), indicating that improved (perceived) workflow compatibility of the interventions is related with a higher intention to use. ¬RTC was also positively correlated with ITU (r = 0.559), but non-significant. For paper-based interventions PU, PEOU, COM and ¬RTC are all positively correlated with ITU (PU – ITU, r = 0.757; PEOU – ITU, r = 0.914, COM – ITU, r = 0.725, ¬RTC – ITU, r = 0.075), although none of these correlations was significant. For both, computer-based and paper-based measures, self- reported use correlated moderately and insignificantly with ITU (ITU – usageIT, r = 0.560; ITU – usagePoster, r = 0.506; ITU – usageChecklist, r = 0.440).

#### Perceived suitability of interventions for the emergency department (Part B)

**Figure 2:**
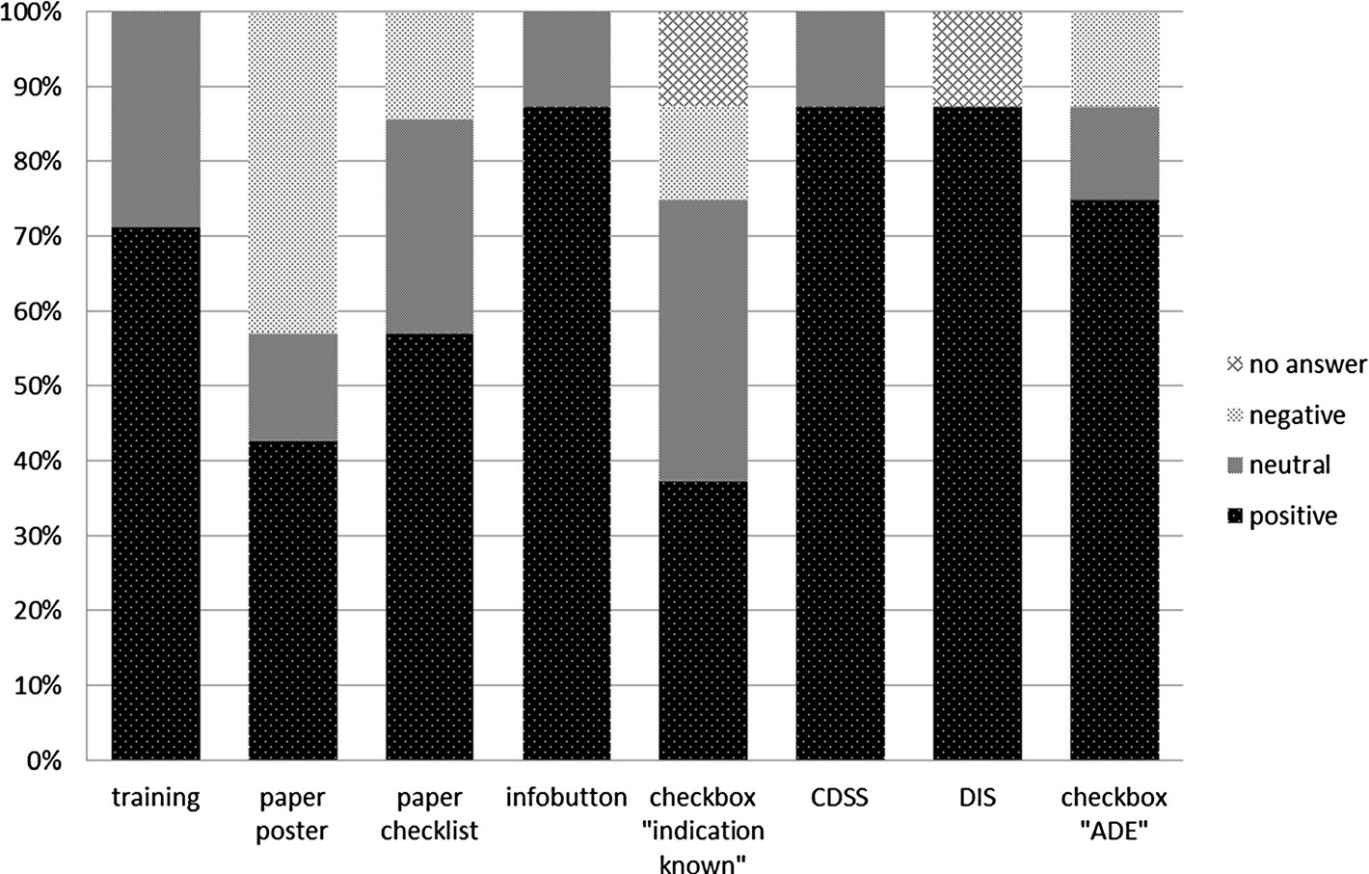
**Rated suitability of the interventions for the emergency department.** Ranking of the interventions based on their relative frequencies. Ratings for “strongly agree” and “agree” were summarized under the category “positive”. Ratings for “strongly disagree” and “disagree” were summarized under the category “negative”.

We asked the participants to assess the suitability of each intervention for the ED environment (item GLO1). There, the repeated medication safety training received the highest rating among the conservative interventions (5/7), followed by the paper-based checklist for physicians’ pocket (4/7) and the paper based posters (3/7). Among the computer-assisted interventions, the infobutton and the “MediCheck” invoking CDSS and DIS were equally rated suitable with seven of eight physicians each. The checkbox “adverse drug event” (ADE) came second (6/8). The checkbox “indication for drug known” was placed last with three of eight cases (see Figure 2). Differences between these rankings, however, were non-significant in Friedman’s test (Chi square: 13.703, p = 0.057) due to small sample size.

## Discussion

### Methods discussion

Our evaluation methodology combined a field observation with a questionnaire-based survey. Field observation is an established method for examining the use of new technologies [41,42]. To determine reasons for (non) acceptance of technology we developed a questionnaire using TAM2 as theoretical framework and extended the model with the factors “resistance to change” and “compatibility” to workflow based on a literature review. Venkatesh and Davis [23], for example, reported that all traditional TAM2 variables influence intention to use (ITU) positively. In addition, studies indicated that “compatibility” (COM) has a positive and direct impact on behavioral intention to use a system [43-45]. Furthermore, Bhattacherjee and Hikmet [39] could show, that users’ “resistance to change” (RTC) is negatively related to their intention to use and that RTC is an important key inhibitor of system utilization. Based on these publications we assumed (for RTC with reversed coding) positive relationships between constructs.

Content validity of the TAM2 part of the questionnaire is based on the fact that all investigated factors reflect the current literature on information technology acceptance in the healthcare domain. Construct validity of this part is based on the fact that only validated constructs of TAM2 [23] and validated scales of previous research [39,40] have been used. We performed a pre-test for the modified questionnaire with nine employees of an independent unit in the University Medical Centre and obtained a Cronbach’s alpha of 0.86 to 1.00 as an indicator for the internal reliability. Nine participants is a comparatively small sample for the pre-test considering the fact that Peleg at al. [46] reported that for eight participants a calculation of reliability was not possible.

### Results discussion

The objectives of this study were (1) to analyze the utilization of different medication safety interventions in a busy ED environment, (2) to assess acceptance of the interventions and their suitability for the ED and (3) to identify reasons for utilization or non-utilization with the goal to derive recommendations for improvement.

Related to our first research question, during the field observation of six times two hours, we did not observe any real live utilization of the implemented medication safety interventions. In comparison, self-reported frequency of use was rather high at least for electronic interventions (daily in five of nine cases) although physicians most often stated that they use the measures only in ten percent of the subjectively critical orders. This seems low; nevertheless there were some physicians who indicated using the interventions regularly. Thus, frequency of usage may depend on the physician. Work experience and expert knowledge could be determining factors where experienced physicians may not have the need to check every prescription. Due to small sample size we are not able to confirm or deny such an assumption. According to self-reported data computer-based interventions have been used more often than paper-based interventions. The infobutton producing a tooltip with short information of general interactions and contra-indications for the respective drug was most used. The discrepancy between no observed factual use of the interventions in the field observation and a comparatively higher self-reported use could have several reasons. The field observation time was limited and may have missed critical patient cases or physicians with a higher use of the interventions. A Hawthorne effect cannot be excluded, which could have resulted in lesser use of interventions than expected. There may be a discrepancy between intention to use and actual use, see below.

Regarding the second research question, results of the questionnaire showed a moderate (paper-based interventions) to rather high (computer-based interventions) degree of acceptance respectively intention to use the medication safety measures in future. Overall, the computer-based interventions tend to be better accepted than the paper-based interventions such as repeated training, checklist and posters which corresponds to the higher self-reported utilization of computer based interventions. However, due to our small sample size these differences are not significant. Zhang et al. [38], Chismar and Wiley-Patton [35] and Yu et al. [37] - who applied TAM2 - have identified perceived usefulness as the most important determinant of acceptance. This is in line with our results, although only significant for computer-based interventions. As physicians were generally positive towards the usefulness of the interventions, an important pre-condition to promote improved medication safety is given.

Concerning the self-rated suitability for the ED, computer-based interventions and the repeated medication safety training received the highest ranking and therefore seem to be the best strategy in the eyes of ED physicians. Paper-based checklists ranked second; posters were judged less appropriate. An assessment of the outcome of these interventions (e.g. reduction of adverse drug events respectively medication errors) will provide a more comprehensive picture of the overall suitability and is planned for future research.

Related to the third research question - reasons for (non) utilization and recommendations for improvement - field observation results indicated that availability and time pressure might be a cause. Clinical work in ED is characterized by many unexpected events and time critical processes. In addition, the physicians move frequently between patients. Hence, interventions must be compatible to the time critical and mobile nature of work and must provide physicians with drug information at all locations. Posters, for example were not visible from all treatment cubicles. The fact that limited availability at the point of care impedes usage behavior was also reported by Rahmner et al. [47]. In contrast to posters, checklists were directly available but were rarely applied, because of physicians’ habit of using the “Arzneimittel pocket” (a German pocket drug reference). Free comments in the questionnaire indicated, that the checklist containing only a small number of critical drugs may have been too specific. All computer-based interventions required previous manual documentation of all drugs into the electronic case sheet. Field observation (and log data) confirmed lack of time to perform this tedious task. This is similar to other studies, which reported lack of time as important drawback for physicians [48-50]. As an indicator for a missing fit to workflow we observed the need for workarounds to document half or quarter tablet regimens.

TAM2 scales showed consistently that medication safety interventions – despite being judged useful and of reasonable information quality – were assessed negatively regarding ease-of-use and compatibility to existing work procedures. Similar to us, other studies reported, that ease-of-use remains a main barrier for rejection [51,52] and that “fit” into the clinical work environment is an important factor for adopting innovations [48]. Free comments in the questionnaire indicated a lack of promotion and briefing for interventions, which we believe is an additional reason for low utilization. Support for this conclusion is offered by Avery et al. [51] and Patterson et al. [53], who identified system training as significant factor contributing to physicians’ use. In addition, Aydin and Forsythe [54] found that physicians “must be comfortable enough with the system to use it in patient’s presence”. Wu et al. [43] have shown that training programs and support will enhance physicians’ confidence and increase perceptions of ease-of-use and usefulness.

From these results we derive several recommendations for enhancing acceptance and usage of medication safety interventions in ED, which are:

● to emphasize the potential benefits of such interventions,

● to establish an automated drug documentation as a prerequisite electronic medication safety interventions. This could be realized with a transfer of medication data from the future electronic health insurance card,

● to signal feedback regarding the utilization of medication safety interventions, e.g. in meetings and training sessions and

● to give physicians more time to adapt to new ways of working.

To what extent the findings of this study and its recommendations will actually lead to a higher level of acceptance and more frequent utilization remains an interesting question for a follow-up study.

Consolidating the results, our findings indicate a gap between usage and intention to use: Paper-based and computer-based interventions in general are accepted, but physicians do not systematically use them. This is consistent to other studies that demonstrated that although physicians developed an intention to use IT, they did not use it [55-57]. Among the reasons we identified lack of access, missing briefing for the interventions, inappropriate ease-of-use, missing compatibility to existing workflows and lacking time for familiarization (see research question 3). The TAM2 items for intention to use refer to “if barriers would have been overcome”. In our case, physicians are willing to apply the interventions but refrain because of these barriers.

For computer-based intervention we found a positive relationship between perceived usefulness (PU) respectively compatibility (COM) and the intention to use the interventions in future (PU – ITU, r = 0.960, p < 0.05; COM – ITU, r = 0.917, p < 0.05). Therefore, addressing these factors is important for reaching a high acceptance of medication safety interventions. With 0.960 the PU – ITU value is above those of Venkatesh and Davis [23], Heselmans et al. [58] and Tung et al. [44] who measured correlation coefficients between 0.44 and 0.73, but this result must be considered cautiously in view of the small sample size. In general, with many correlation coefficients being non-significant even in the range of 0.7 to 0.8, the sensitivity of our study to detect other, potentially relevant correlations is admittedly low. Nevertheless, our results are comparable to TAM2 findings of previous health IT research [35-38] and indicate the applicability of TAM2 for a busy ED environment.

### Implications for practice and research

We could demonstrate that TAM2 is a useful diagnostic instrument to understand usage of medication safety measures. Our results may help identifying strategies and focusing tasks for enhancing acceptance. Implications for further research arise from the fact that the TAM2 was developed outside of clinical environments and therefore some of the items do not meet ED requirements. To measure e.g. subjective norm as social influence of the opinion of other physician colleagues may not be meaningful in the ED context, because sources of social influence may rather be department heads or nursing staff. Although (modified) TAM2 comprises variables that are related to usage intention this does not mean that these variables are all-embracing. There may be other factors such as trust, accessibility, visible management support, that could serve as barriers to ED physician’s adoption. A challenge is to identify and to test these alternative factors in order to develop a further improved and more contextualized version of TAM2.

### Limitations of the study

The application oriented context of this study required pragmatic solutions at various points. The study was conducted in one ED only, where specific interventions to improve medication safety had been implemented. Use of the interventions and of electronic documentation was voluntary. Findings cannot be generalized to other settings and other interventions without further research. The field observation examined only a small proportion of physicians working at the ED and a small patient sample. Due to anonymization we could not testify the real need for a medication check for each of those patients. The questionnaire survey included 75 percent of the candidates working permanently in the ED, but not those physicians from other departments working occasionally in night shifts and weekends in the ED. The questionnaire sample was small rendering statistical work-up difficult, thus the results must be interpreted with caution. To detect relationships between TAM2 variables we used Pearson’s score which assumes metrical data. This is disputable from a statistical point of view, but our approach makes the study comparable to other TAM based research [45,58]. Further, participation of nine of eligible 12 ED physicians in the TAM assessment may indicate some volunteer bias. Due to the sample size the TAM2 model assumptions could not be calculated, therefore additional research is indicated to determine the degree of influence of the different acceptance factors on system utilization. We relied on the TAM2 variables and did not assess other potentially relevant variables such as trust in the provided information, computer self-efficacy, or the availability of organizational or technical support. Ideally an acceptance study takes place during system development and is repeated after rollout of the (improved) system. In comparison, this study examined utilization and acceptance retrospectively with no direct impact of its results to improve the implemented medication safety interventions.

### What this study adds to knowledge

Although the TAM model has been applied across a number of health contexts and technologies [26,27] only few studies related to medication safety [15,59], CDSS [46,58] or the ED context [60] could be identified. To our knowledge, TAM2 has not been previously applied in the ED area making this one of the first studies of its kind. Also new is the extension of TAM2 with the factors “resistance to change” and “compatibility” which allowed additional insight into ED physicians’ behavioral intention and actual usage.

We could show that physicians preferred especially the medication safety training and computer-assisted safety checks. Computer-assisted interventions (infobutton, CDSS and DIS) tend to be better accepted compared to paper-based interventions, despite their still low ease-of-use and workflow fit.

## Conclusions

The aim of this study was to evaluate physicians’ use and acceptance of different interventions in an ED and to identify reasons why interventions are adopted or rejected. We found it critical that, despite their positive attitude, physicians reported use of interventions in only ten percent of critical orders which correlates to our observation results. Thus we recommend increased attention to the main barriers identified in our study which include insufficient access, insufficient briefing for the use of interventions, insufficient compatibility to workflow, lacking ease-of-use and lacking time for familiarization. We encourage other EDs to assess medication safety interventions from a multi-factor perspective, not only focusing on technical aspects, but also on change management.

## Abbreviations

CDSS: Clinical decision support systems; COM: Compatibility; cPOE: Computerized drug order entry, DDI, Drug-drug interactions; DIS: Drug information system; ED: Emergency department; EHR: Electronic health record; IM: Image; ITU: Intention to use; JR: Job relevance; ME: Medication error; SN: Subjective norm; OQ: Output quality; PEOU: Perceived ease-of-use; PU: Perceived usefulness; RD: Result demonstrability; RTC: Resistance to change; TAM: Technology acceptance model; VO: Voluntariness.

## Competing interests

The authors declare that they have no competing interests.

## Authors’ contributions

HD, RM, and TB designed the underlying BMG sponsored study to develop and evaluate measures for medication safety. MK, AS, FM, BP, BPK and RV collected data for the underlying study. BS and TB designed the observational and TAM based assessment of measures. BS and HD contributed to the data acquisition, analysis and interpretation of data. BS has written the first draft of the article and has edited the final version. HD, RM, TB, HUP and MCR have contributed to the article by revising the first draft and providing various comments. All authors read and approved the final manuscript.

## Pre-publication history

The pre-publication history for this paper can be accessed here:

http://www.biomedcentral.com/1472-6947/13/79/prepub

## Supplementary Material

Additional file 1 **Survey Instrument.** Questionnaire used to collect data from ED physicians on usage and acceptance of different medication safety measures. Click here for file
