# Supplementary material for: Comparative evaluation of different medication safety measures for the emergency department: physicians’ usage and acceptance of training, poster, checklist and computerized decision support
Source: BMC Med Inform Decis Mak. 2013 Jul 29;13:79. doi: 10.1186/1472-6947-13-79 (PMC3733614; doi:10.1186/1472-6947-13-79)
Supplement: Additional file 1 — Survey Instrument. Questionnaire used to collect data from ED physicians on usage and acceptance of different medication safety measures. [file 1472-6947-13-79-S1.pdf]

Fragebogen zur Bewertung von AMTS- Maßnahmen (ZNA, Fürth)

Liebe Ärztin, lieber Arzt,

in der ZNA des Klinikums Fürth wurden vor einiger Zeit verschiedene **Maßnahmen zur Verbesserung der Arzneimitteltherapiesicherheit** eingeführt. Diese Einführung wird von uns wissenschaftlich begleitet. Mit dem vorliegenden Fragebogen möchten wir Ihre **Ansichten und Einstellungen** zu diesen Maßnahmen ermitteln. Wir möchten Sie deshalb bitten, sich etwas Zeit zu nehmen und den Fragebogen zu beantworten.

Die Fragen beziehen sich dabei vor allem auf

- die **Schulungen** in der Montagsbesprechung
- die in der ZNA aufgehängten **Informationsposter**
- die **Medikamenten- Checkliste** für die Kitteltasche sowie
- die **Erweiterungen des elektronischen Krankenblattes** um den **Info-Knopf** (erscheint neben den eingetragenen Medikamenten), die Felder **„Indikation bekannt“** und **„UAE“** (unerwünschtes Arzneimittelereignis) sowie den Knopf **„MedikationsCheck“** (unterhalb der Medikamentenliste; dient dem Abruf von Kontraindikationen und Interaktionen in OntoDrug sowie dem Abruf ausführlicher Fachinformationen in PharmIndex Plus).

Alle Daten werden selbstverständlich **anonym** erhoben, so dass keine Ihrer Aussagen auf Sie persönlich zurückgeführt werden kann. **Bitte antworten Sie ehrlich und lassen Sie möglichst keine der Fragen aus.** Bei Rückfragen wenden Sie sich bitte an die Person, die diesen Fragebogen ausgeteilt hat.

Herzlichen Dank für Ihre Mitarbeit!
Ihr AMTS- Studien- Team

Teil A: Fragen zur Nutzung der verschiedenen Arzneimitteltherapiesicherheits- (AMTS-) Maßnahmen

Im Folgenden finden Sie eine Reihe von Aussagen zur Nutzung der verschiedenen AMTS- Maßnahmen. Bitte kreuzen Sie die jeweils zutreffende Antwort an oder füllen Sie die Leerstellen aus.

U1.	Wenn Sie Fragen zu Kontraindikationen und Interaktionen von Medikamenten haben, wie informieren Sie sich **im ersten Schritt**? (Bitte nur eine Antwort ankreuzen.)	☐ ich schaue auf das Informationsposter<br>☐ ich schaue auf die Checkliste meiner Kitteltasche<br>☐ ich führe einen elektronischen Medikations-Check durch<br>☐ ich frage eine Arbeitskollegin/ einen Arbeitskollegen<br>☐ ich schaue in der „Roten Liste“ (Printversion) nach<br>☐ Sonstiges (bitte ergänzen): ________________________________
U2.	Wurde Ihnen die Benutzung der folgenden AMTS- Maßnahmen vorab erklärt?	Poster ☐ ja ☐ nein<br>Checkliste ☐ ja ☐ nein<br>Elektronischer Medikations-Check ☐ ja ☐ nein
U3.	Wie oft nutzen Sie die folgenden AMTS- Maßnahmen im Alltag?	Poster ☐ nie ☐ monatlich ☐ wöchentlich ☐ 1x täglich ☐ mehrmals täglich<br>Checkliste ☐ nie ☐ monatlich ☐ wöchentlich ☐ 1x täglich ☐ mehrmals täglich<br>Elektronischer Medikations-Check ☐ nie ☐ monatlich ☐ wöchentlich ☐ 1x täglich ☐ mehrmals täglich
U4.	**Bei problematischer Arzneiverordnung**: Bei wie viel Prozent der von Ihnen durchgeführten Verordnungen verwenden Sie jeweils die folgenden AMTS- Maßnahmen?	Poster ☐ nie ☐ bis 10% ☐ bis 25% ☐ bis 50% ☐ bis 100%<br>Checkliste ☐ nie ☐ bis 10% ☐ bis 25% ☐ bis 50% ☐ bis 100%<br>Elektronischer Medikations-Check ☐ nie ☐ bis 10% ☐ bis 25% ☐ bis 50% ☐ bis 100%
U5.	Wann haben Sie die folgenden AMTS- Maßnahmen das letzte Mal benutzt?	Poster ☐ noch nie benutzt ☐ heute ☐ vor _____ Tagen<br>Checkliste ☐ noch nie benutzt ☐ heute ☐ vor _____ Tagen<br>Elektronischer Medikations-Check ☐ noch nie benutzt ☐ heute ☐ vor _____ Tagen
U6.	Auf welche der folgenden Informationsquellen greifen Sie in der Regel bei Nutzung des elektronischen Medikations-Checks zurück? (Mehrfachauswahl möglich.)	☐ Info-Knopf „i“ mit Tooltip (erscheint neben den eingetragenen Medikamenten)<br>☐ das Feld „Indikation bekannt“<br>☐ OntoDrug (Informationen zu Kontraindikationen und Interaktionen)<br>☐ PharmIndex Plus (ausführliche Fachinformationen)<br>☐ das Feld „UAE“ (unerwünschtes Arzneimittelereignis)<br>☐ auf keine der genannten Informationsquellen

U7.	In welchen Situationen (wann) nutzen Sie den elektronischen Medikations- Check? (stichpunktartig)<br>__________<br>__________<br>__________
U8.	Bei ersten Beobachtungen ist aufgefallen, dass die Maßnahmen Poster, Checkliste und elektronischer Medikations-Check eher selten genutzt werden. Welche Gründe könnte es Ihrer Meinung nach dafür geben? (stichpunktartig)<br>Mögliche Gründe für die seltene Nutzung von Poster und Checkliste:<br>__________<br>__________<br>__________<br>Mögliche Gründe für die seltene Nutzung des elektronischen Medikations-Checks:<br>__________<br>__________<br>__________
U9.	Was müsste Ihrer Meinung nach getan werden, damit die AMTS-Maßnahmen Poster, Checkliste sowie elektronischer Medikations-Check häufiger genutzt werden? (stichpunktartig)<br>__________<br>__________<br>__________

Teil B: Fragen zur Akzeptanz der verschiedenen Arzneimitteltherapiesicherheits- (AMTS-) Maßnahmen

Im Folgenden finden Sie eine Reihe von Aussagen zur Akzeptanz der durchgeführten AMTS- Maßnahmen.

Der **erste Zeile jeder Aussage** bezieht sich jeweils auf die **papierbasierten und Schulungsmaßnahmen**, d.h. konkret die **Schulungen („S“)** in der Montags- Frühbesprechung, die aufgehängten **Poster („P“)** zur AMTS und die Medikamenten- **Checkliste („C“)** für die Kitteltasche.

Der **zweite Zeile jeder Aussage** bezieht sich jeweils auf alle **EDV- basierten Maßnahmen („EDV“)** die im elektronischen Krankenblatt in ORBIS umgesetzt wurden, d.h. die **Ankreuzfelder** für **„Indikation bekannt“** und **„UAE“** (unerwünschtes Arzneimittelereignis), die **Tooltipps**, die bei dem „i“ () für die einzelnen Medikamente erscheinen, die Möglichkeit, über den **„MedikationsCheck“ -Button** Kontraindikationen und Interaktionen zu prüfen und die Möglichkeit, von dort aus in PharmIndex Plus weitere Fachinformationen zum Medikament abzufragen.

Bitte schätzen Sie Ihre Zustimmung zu jeder Aussage auf der daneben befindlichen Skala von „stimmt überhaupt nicht“ bis „stimmt völlig“ ein. Für den Fall, dass Sie sich aus irgendwelchen Gründen nicht dazu äußern wollen oder können oder die jeweiligen Maßnahmen nicht genutzt haben, kreuzen Sie bitte „keine Angabe“ an.

			stimmt **überhaupt nicht**	stimmt **eher nicht**	stimmt **teils/ teils**	stimmt **eher**	stimmt **völlig**	**keine Angabe**
PU1.	Die in der ZNA eingeführten AMTS-Maßnahmen zur Verbesserung der Arzneimitteltherapiesicherheit halte ich grundsätzlich für sinnvoll.	S/P/C	☐	☐	☐	☐	☐	☐
		EDV	☐	☐	☐	☐	☐	☐
PU2.	Die AMTS- Maßnahmen können mein Arbeitsergebnis verbessern.	S/P/C	☐	☐	☐	☐	☐	☐
		EDV	☐	☐	☐	☐	☐	☐
PU3.	Die AMTS- Maßnahmen können meine Qualität der Versorgung verbessern.	S/P/C	☐	☐	☐	☐	☐	☐
		EDV	☐	☐	☐	☐	☐	☐
PU4.	Die AMTS- Maßnahmen können meine Effektivität erhöhen.	S/P/C	☐	☐	☐	☐	☐	☐
		EDV	☐	☐	☐	☐	☐	☐
PU5.	Die AMTS- Maßnahmen können für meine Arbeit nützlich sein.	S/P/C	☐	☐	☐	☐	☐	☐
		EDV	☐	☐	☐	☐	☐	☐
EoU1.	Mein Umgang mit den AMTS- Maßnahmen ist einfach und verständlich.	S/P/C	☐	☐	☐	☐	☐	☐
		EDV	☐	☐	☐	☐	☐	☐
EoU2.	Die AMTS- Maßnahmen sind einfach anzuwenden.	S/P/C	☐	☐	☐	☐	☐	☐
		EDV	☐	☐	☐	☐	☐	☐

			stimmt **überhaupt nicht**	stimmt **eher nicht**	stimmt **teils/ teils**	stimmt **eher**	stimmt **völlig**	**keine Angabe**
EoU3.	Der Umgang mit den AMTS- Maßnahmen erfordert wenig geistige Anstrengung.	S/P/C	☐	☐	☐	☐	☐	☐
		EDV	☐	☐	☐	☐	☐	☐
EoU4.	Die AMTS- Maßnahmen liefern genau das, was ich von ihnen erwarte.	S/P/C	☐	☐	☐	☐	☐	☐
		EDV	☐	☐	☐	☐	☐	☐
SN1.	Kollegen/-innen, die mein Verhalten beeinflussen, meinen, dass ich die AMTS- Maßnahmen anwenden sollte.	S/P/C	☐	☐	☐	☐	☐	☐
		EDV	☐	☐	☐	☐	☐	☐
IM1.	Über die AMTS- Maßnahmen zu verfügen ist ein Hervorhebungsmerkmal.	S/P/C	☐	☐	☐	☐	☐	☐
		EDV	☐	☐	☐	☐	☐	☐
IM2.	Kollegen/- innen, die die AMTS- Maßnahmen anwenden sind besser angesehen als diejenigen, die die AMTS- Maßnahmen nicht anwenden.	S/P/C	☐	☐	☐	☐	☐	☐
		EDV	☐	☐	☐	☐	☐	☐
IM3.	Kollegen/-innen, die die AMTS-Maßnahmen anwenden, haben eine höhere Bekanntheit.	S/P/C	☐	☐	☐	☐	☐	☐
		EDV	☐	☐	☐	☐	☐	☐
VO1.	Die Anwendung der AMTS-Maßnahmen ist freiwillig.	S/P/C	☐	☐	☐	☐	☐	☐
		EDV	☐	☐	☐	☐	☐	☐
JR1.	Die AMTS- Maßnahmen anzuwenden, ist sachdienlich für die Patientenversorgung.	S/P/C	☐	☐	☐	☐	☐	☐
		EDV	☐	☐	☐	☐	☐	☐
JR2.	Die AMTS- Maßnahmen anzuwenden ist wichtig für die Patientenversorgung.	S/P/C	☐	☐	☐	☐	☐	☐
		EDV	☐	☐	☐	☐	☐	☐
COM	Die Benutzung der AMTS- Maßnahmen passt zu meinem Arbeitsablauf.	S/P/C	☐	☐	☐	☐	☐	☐
		EDV	☐	☐	☐	☐	☐	☐
OQ1.	Die Qualität der fachlichen Informationen der AMTS- Maßnahmen ist hoch.	S/P/C	☐	☐	☐	☐	☐	☐
		EDV	☐	☐	☐	☐	☐	☐
RD1.	Die AMTS- Maßnahmen können die Kosten meiner medizinischen Versorgung senken.	S/P/C	☐	☐	☐	☐	☐	☐
		EDV	☐	☐	☐	☐	☐	☐
RD2.	Ich glaube, ich kann Anderen die Auswirkungen der AMTS- Maßnahmen vermitteln.	S/P/C	☐	☐	☐	☐	☐	☐
		EDV	☐	☐	☐	☐	☐	☐
RD3.	Die Auswirkungen der AMTS- Maßnahmen sind für mich offensichtlich.	S/P/C	☐	☐	☐	☐	☐	☐
		EDV	☐	☐	☐	☐	☐	☐
RD4.	Ich habe keine Schwierigkeiten zu erklären, warum die Verwendung der AMTS- Maßnahmen nützlich ist.	S/P/C	☐	☐	☐	☐	☐	☐
		EDV	☐	☐	☐	☐	☐	☐
RtC1.	Ich möchte nicht, dass die AMTS- Maßnahmen die Art verändern, wie ich Medikamente verordne.	S/P/C	☐	☐	☐	☐	☐	☐
		EDV	☐	☐	☐	☐	☐	☐
RtC3.	Alles in allem möchte ich nicht, dass die AMTS- Maßnahmen die Art verändern, wie ich aktuell arbeite.	S/P/C	☐	☐	☐	☐	☐	☐
		EDV	☐	☐	☐	☐	☐	☐
ItU1.	Sobald die erheblichen Hürden für die Nutzung überwunden sind, plane ich die AMTS- Maßnahmen zu nutzen.	S/P/C	☐	☐	☐	☐	☐	☐
		EDV	☐	☐	☐	☐	☐	☐
ItU2.	Wenn die erheblichen Hürden für die Nutzung nicht existieren würden, würde ich die AMTS- Maßnahmen nutzen.	S/P/C	☐	☐	☐	☐	☐	☐
		EDV	☐	☐	☐	☐	☐	☐

	Für wie geeignet halten Sie die folgenden AMTS-Maßnahmen?	überhaupt nicht geeignet	eher nicht geeignet	teilweise geeignet	eher geeignet	völlig geeignet	keine Angabe
EIG1.	Schulungen	☐	☐	☐	☐	☐	☐
EIG2.	Informationsposter	☐	☐	☐	☐	☐	☐
EIG3.	Medikamenten- Checkliste für die Kitteltasche	☐	☐	☐	☐	☐	☐
EIG4.	Info- Knopf „i“ mit Tooltip (im elektronischen Krankenblatt)	☐	☐	☐	☐	☐	☐
EIG5.	das Feld „Indikation bekannt“ (im elektronischen Krankenblatt)	☐	☐	☐	☐	☐	☐
EIG6.	OntoDrug- Informationen zu Kontraindikationen und Interaktionen (im elektronischen Krankenblatt)	☐	☐	☐	☐	☐	☐
EIG7.	PharmIndex Plus- ausführliche Fachinformationen (im elektronischen Krankenblatt)	☐	☐	☐	☐	☐	☐
EIG8.	das Feld „UAE“- unerwünschtes Arzneimittelereignis (im elektronischen Krankenblatt)	☐	☐	☐	☐	☐	☐

Ein Fragebogen kann nicht alle Aspekte ansprechen. Dieser Teil des Fragebogens ist für Ihre individuellen Anmerkungen reserviert. **Möchten Sie uns noch auf etwas aufmerksam machen?** (stichpunktartig)

____

____

____

____

Teil C: Fragen zur Person- Diese Angaben sind freiwillig!

Abschließend bitten wir Sie noch, uns einige Fragen zu Ihrer Person zu beantworten. **Alle Angaben sind freiwillig**, sie unterstützen uns aber bei der statistischen Auswertung dieses Fragebogens.

DG3.	Zu welcher Berufsgruppe gehören Sie?	☐ Oberarzt/-ärztin ZNA<br>☐ Facharzt/-ärztin ZNA<br>☐ Assistenzarzt/-ärztin ZNA		☐ Facharzt/-ärztin Med1/Med2<br>☐ Assistenzarzt/-ärztin Med1/ Med2<br>☐ AiP	
EXP1.	Wie lange arbeiten Sie schon als Arzt/ Ärztin (mit AiP)?	seit _____ Jahren			
EXP2	Wie lange arbeiten Sie schon mit elektronischen Patientenakten?	seit _____ Jahren			
EXP3.	Wie schätzen Ihre Computerkenntnisse im Allgemeinen ein?	☐ gering	☐ mittelmäßig	☐ hoch	
KENN	Bitte bilden Sie sich Ihr individuelles Kennwort (Aus Datenschutzgründen, statt Name):	____<br>Anfangsbuchstabe Ihres Geburtsortes	____<br>Endbuchstabe des Vornamens Ihrer Mutter	____<br>Anfangsbuchstabe des Vornamens Ihrer Mutter	____<br>Endbuchstabe Ihres eigenen ersten Vornamens

Wir bedanken uns sehr herzlich für Ihre Mühe und Zeit!
